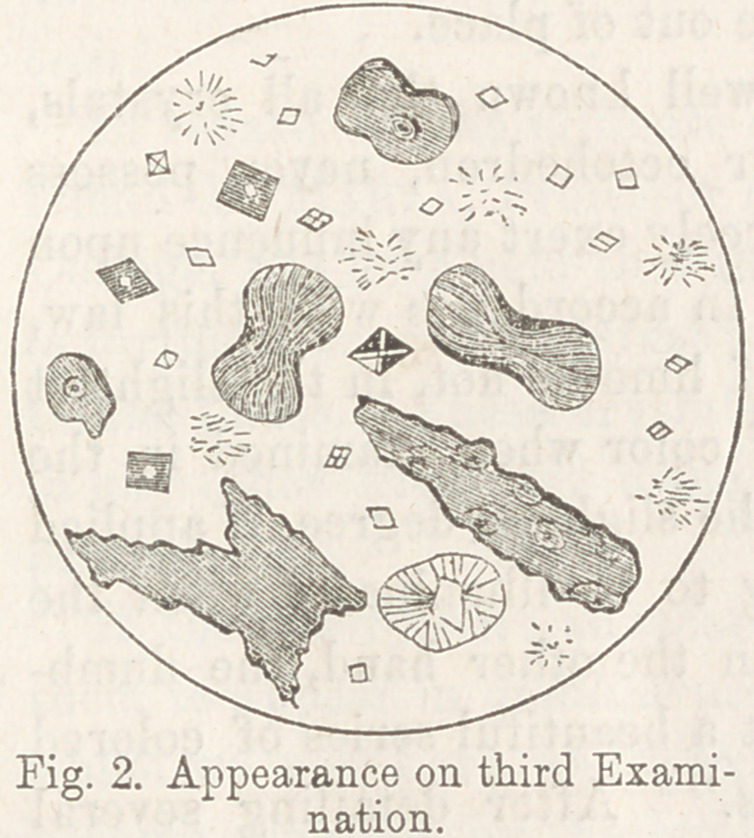# A New Theory of the Origin of “Dumb-Bell” Crystals

**Published:** 1866-05

**Authors:** D. W. Flora

**Affiliations:** Cottage Grove Avenue; Chicago, Ill.


### CHICAGO MEDICAL EXAMINER.

N. S. DAVIS, M.D., Editor.

VOL. VII. MAY, 1866. NO. 5.

### Original Contributions.

### ARTICLE XVI.

### A NEW THEORY OF THE ORIGIN OF "DUMB-BELL" CRYSTALS.

By D. W. FLORA, M.D., Chicago, Ill.

It is proposed to open the discussion, by giving the history of a case which fell under my treatment, about one year ago, in which the urine of the patient contained abundance of the crystals in question, in common with the oxalates of lime.

The observations made in this case from day to day, led me to doubt the existence of the "dumb-bell" as a primary form of crystallization, and to ascribe its occasional appearance to mere accident depending upon changes produced by the accidental union of other crystals.

### CASE OF DYSPEPSIA WITH OXALURIA.

S. C., *æt.* 32 years, a private of Co. F., 13th Mich. Volunteer Infantry, was admitted to Madison U.S. General Hospital Dec. 1st, 1864. On admission, the patient appeared somewhat cachectic, and had dyspeptic symptoms, with obstinate constipation. ℞. Pil. cath. comp., No. iij., to be followed by Seidlitz pulv. every hour, till bowels move. To keep the bowels in a soluble state, fl. ext. rhei, ℨj. was ordered daily.

*Dec. 12th.*—Patient seized with a tertian ague, which readily yielded to sul. quinine. An ulcer on the left leg, over the spine of the tibia, was noticed about this time to be in an indolent condition. This ulcer occupied the site of a previous bruise.

*Dec. 19th.*—Constipation still continues, and the ulcer inclines to spread. The spittoon by the patient's bed was observed to be filled with rejected food after meals, which the patient stated was thrown from the stomach immediately after being swallowed. He was taxed with voluntary vomiting, which he stoutly denied.

*Jan. 20th, 1865.*—The vomiting still continues. He was put upon raw beefsteak, cut into fine cubes, ℥iij. daily. No fluids allowed. *Jan. 30th.*—No change. Smoked ham was then substituted for the raw beef, but with no better results. *Feb. 1st.*—About this time, the patient complained of pain and tenderness in the renal region, with frequent desire to micturate. The urine was rather abundant, of a deep straw color, or light gamboge. The reaction was slightly acid—sp. gr. 1022. On cooling, a dense precipitate was formed, equal to one-fourth the whole bulk. This cleared up under the influence of heat, and also readily on the addition of nitric acid. A drop placed upon a glass slide, and allowed to crystallize spontaneously, presented the appearance seen below. The *urates* were undoubtedly present, as were also deposits of epithelium and uriniferous casts. The octohedral crystals of oxalate of lime were seen in myriads, many of them exceedingly minute.—Stellate crystals, composed of numerous fine prisms arranged around a common centre, were also numerous, as may be seen by reference to the drawing. A large cruciform crystal was occasionally to be seen in the field. "*Dumb-bells*" *were sought for in vain!* The stellate crystals were undoubtedly *urates*,

Fig. 1. Appearance on first Examination.

but whether of ammonia, soda, or potash, I was, at the time, unable to make out.

These stellates are the crystals which are hereafter to figure as "dumb-bells." This first specimen was carefully set aside, still under the field of the microscope, and twenty-four hours afterwards a *second* examination was made, when the stellate crystals, composed of the urates, showed a tendency to unite by solution or liquefaction of a portion of their discs. This is the *first step* in the formation of "dumb-bells," and it requires only a slight modification to complete the metamorphosis. By the pressure of fluids (for at this time rapid deliquescence of the crystals is taking place) upon the outside of the now united discs, the rim is forced inward upon itself at the point of least resistance, to wit:—at the point of junction of the two discs the radii being already dissolved at their points of contact. The prisms which formed the radii of the circles are now set afloat, and arrange themselves parallel to each other and in the direction of the long diameter of the "dumb-bell."

When examined again, say 30 hours after the first, the long-sought "dumb-bell" was discovered in the same field which had been twice explored unsuccessfully. [See Fig. 2.]

Fig. 2. Appearance on third Examination.

Such, in brief, is the history of the case which led to a series of investigations, and the adoption of the theory of "dumb-bells," which it is the object of this paper to elucidate. It may be added, that this man was discharged from the service of the Government four months after admission, no improvement having been observed in his condition. Before leaving the hospital, he boasted to his nurse that he "had played his game successfully, as his vomiting had all been produced voluntarily." If his admission be true, it is a remarkable case, exhibiting all the aggravated symptoms of *dyspepsia*

as cachexia, oxaluria, etc., brought upon himself and maintained during a period of four months, by persistent voluntary rejection of his food.

I shall take the liberty to refer to a case now under treatment, in which the urine is acid when first examined, and contains the *urates*, as well as the *oxalates*, in great abundance. The deposits have been more thoroughly and carefully examined than in the preceding case, and have given almost precisely similar results. On the fourth or fifth day, a final examination was made, the results of which very nearly resembled the appearance figured in Dr. GOLDING BIRD's work on Urinary Deposits, in which he intends to represent one phase of the "dumb-bell" crystals. The forms referred to appeared at a time, and under circumstances, which leave no doubt on my mind that they are the last which these crystals (dumb-bells) take, previous to their entire liquefaction.

Although it is not incumbent on me to prove the exact composition of these crystals in question, in order to sustain my theory, yet a brief resumé of the opinions of some prominent authors on this subject may not be out of place.

GOLDING BIRD says:—"It is well known that all crystals, referable to the cube or regular octohedron, never possess double refraction, and, hence, scarcely exert any influence upon a plain polarized ray of light. In accordance with this law, the ordinary crystals of oxalate of lime do not, in the slightest degree, exhibit the phenomena of color when examined in the polarizing microscope, merely in the slightest degree, if applied in a favorable position, appearing to be illuminated when the polarizing prisms are crossed. On the other hand, the dumb-bells, as I long ago stated, exhibit a beautiful series of colored rings traversed by a black cross." After detailing several experiments with these dumb-bells, he says:—"We may safely conclude that they do not consist of mere oxalate of lime, for their powerful action on polarized light is quite incompatible with their being composed exclusively of this salt. The action of heat shows that they are readily converted into carbonate of lime without change of form." In conclusion, the same author says:—"I think we may venture to assume the high probability of these crystals (dumb-bells) consisting of the oxalurate of lime."

Dr. HASSALL, in the *British and Foreign Medico-Chirurgical Review*, remarks:—"That soluble dumb-bells in the urine frequently consist of sulphuric acid in combination with soda or potash."

Dr. OTTO FUNKE, in his beautiful micographic work on urinary deposits, has figured and described these crystals as composed of the urates of soda.

Dr. BACON, in the *American Journal of Medical Sciences*, for April, 1851, is inclined to regard the oval crystals shown in my last drawing, as "dumb-bells seen endwise." He dissolved them in strong acetic acid, and on spontaneous evaporation they presented abundance of zeolitic crystals, from "*circular striated plates* to *dumb-bells*."

This experiment strengthens the position taken in this essay, for unless this "zeolitic arrangement" is present, unless we have the "circular striated crystals," we *cannot have dumb-bells!*

In regard to the ultimate composition of the dumb-bell, my friend F. MAHLA, Professor of Chemistry in Chicago Medical College, has long held that they are *not* oxalate of lime, and he is further inclined to refuse them a place among *primary* crystalline forms. These crystals have never been observed by me in any other than *acid* urine, in which *urates* were undeniably present.

If the theory here advocated be correct, it is impossible for them to appear in *alkaline* urine, and this is verified in the case which we now have under examination. As soon as the urine became alkaline, by the evolution of ammonia, (the octohedral crystals of oxalate of lime being still present,) the "dumb-bells" disappeared altogether, and were replaced by a copious deposit of the *triple phosphates*.

There are six different geometrical forms to which *all* crystals may be referred, and it would require a great stretch of the imagination to perceive any similarity in the "dumb-bell" to *any* of these forms or their modifications.

The "circular or stellate" crystals, which we regard as the originators of the "dumb-bell," are themselves *secondary* forms, the result of an arrangement of needle-shaped prisms around a common centre. From whence it follows that our famous "dumb-bell," about whose composition there has been so much discussion, and such wide differences of opinion, is only an *accidental* and *tertiary* form, the result of *accident* merely!

*Cottage Grove Avenue.*

## Figures and Tables

**Fig. 1. f1:**
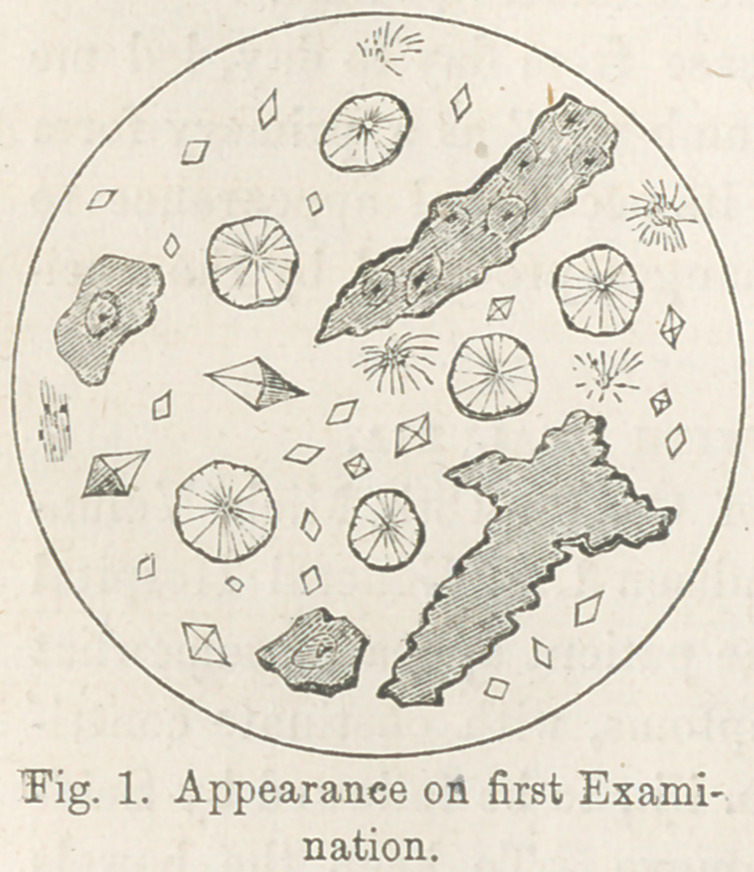


**Fig. 2. f2:**